# Implications of Green Tea and Its Constituents in the Prevention of Cancer via the Modulation of Cell Signalling Pathway

**DOI:** 10.1155/2015/925640

**Published:** 2015-04-21

**Authors:** Arshad H. Rahmani, Fahad M. Al shabrmi, Khaled S. Allemailem, Salah M. Aly, Masood A. Khan

**Affiliations:** ^1^Department of Medical Laboratories, College of Applied Medical Sciences, Qassim University, Buraidah 6699, Saudi Arabia; ^2^Department of Pathology, Faculty of Veterinary Medicine, Suez Canal University, Ismailia, Egypt; ^3^Department of Basic Health Science, College of Applied Medical Sciences, Qassim University, Buraidah 6699, Saudi Arabia

## Abstract

Green tea is commonly used as a beverage worldwide, especially in China, Japan, Morocco, and Saudi Arabia. Green tea and its constituents have been considered very effective in the prevention and treatment of various diseases. It contains a variety of catechins, which show a pivotal role in the modulation of biological activities and also act as chemopreventive agents. Earlier studies have confirmed that green tea and its chief constituent epigallocatechin gallate (EGCG) have a potential role in the management of cancer through the modulation of cell signaling pathways. In this review, we focused on the beneficial effects of green tea and its constituents in the cancer prevention and treatment and its impact on modulation of molecular pathways.

## 1. Introduction

Natural products, mainly plants and their constituents, have been used in the diseases cure from the ancient time and its role in the health management is very popular in India, China, and other parts of the world. In Arab world, natural product/plants seeds and fruits are commonly used in diseases treatment and Prophet Mohammad (PBUH) also suggested various plants such as dates fruits, olive fruits, and black seed in the treatment of diseases [1]. Earlier study revealed that medicinal plants and their ingredients such as olive fruits/oil, dates fruits, and* Nigella sativa* have role as anticancer via modulation of various biological activities [2–5]. However, green tea is a product made from the* Camellia sinensis* plant and is commonly used as beverage worldwide. From the ancient time, green tea and its constituents show role in health management via modulation of biological process including molecular and biochemical pathways. Green tea shows health promoting effects mainly due to the polyphenol content [6], especially flavonols, which constitutes 30% of fresh leaf dry weight [7]. The chief constituents of green tea are catechins where (−)-epigallocatechin gallate is one of the most effective types of catechins. The anticancer effects of (−)-epigallocatechin gallate have been reported via modulation of signaling pathways and also play a role in the downregulation of proteins expression involved in the invasiveness of cancer cells [8]. Another finding has shown that EGCG inhibited growth of the mouse viral mammary epithelial carcinogenesis model; induced apoptosis and finding suggest the clinical relevance of EGCG as a chemopreventive agent [9]. An important study revealed that EGCG, a chief constituent of green tea, significantly reduced tumor volume in xenograft mouse model breast cancer cells [10] and a study summarized the role of the green tea constituents EGCG in chemoprevention [11]. In this review, we focused on the therapeutic role of green tea and its constituents in the management of cancer through modulation of various cell signalling pathways.

## 2. Chemical Structure of Active Constituents of Green Tea

**Figure 1:**
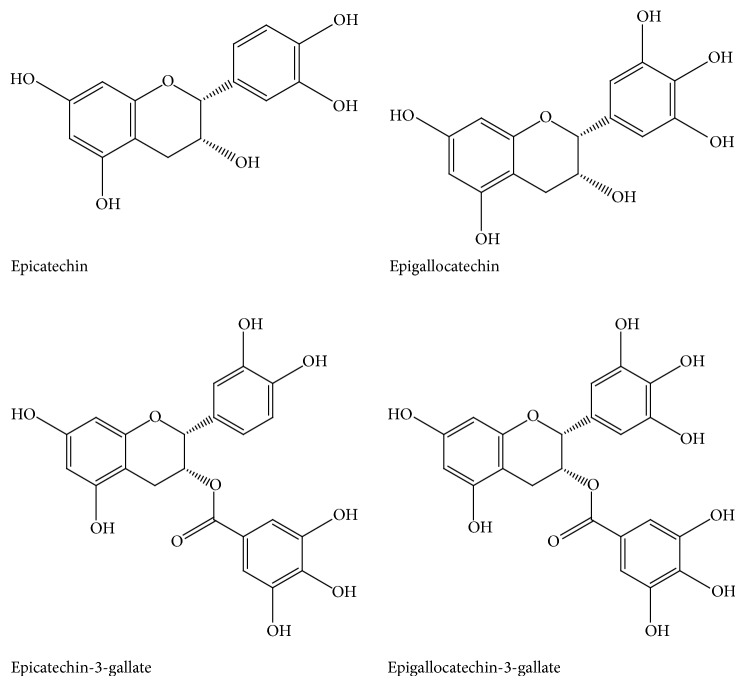
Active constituents of green tea.

Green tea is a complex mixture of precious compounds including polyphenols, flavonoids, flavonols, and other constituents such as amino acids, organic acids, lipids, vitamins, polysaccharides, and thiamine. Catechins are a type of polyphenol and are the main astringency component in green tea and modulate the various genes involved in the development and progression of cancer. The chief catechins are (−)-epicatechin (EC), (−)-epicatechin-3-gallate (ECG), (−)-epigallocatechin (EGC), and (−)-epigallocatechin-3-gallate (EGCG) [12–15]. Approximately 30–42% of the dry weight of green tea contains phenolic compounds [12, 16] and EGCG is one of the most abundant catechins that contains around 50–80% of the total catechins [16]. The chemical structure of chief constituents of green tea is presented in Figure 1.

## 3. Mechanism of Action of Green Tea in Cancer Prevention

**Figure 2:**
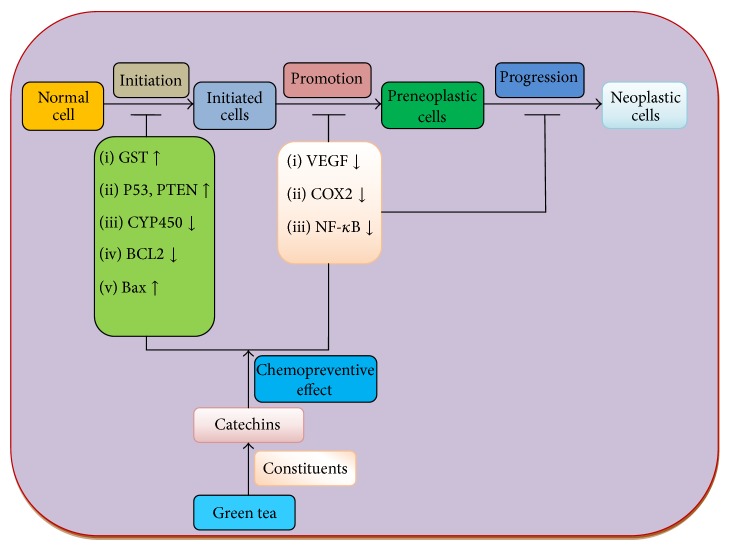
Role of green tea in cancer prevention through the modulation of genes involved in initiations, promotion, and progression of cancer.

Green tea is a product made from the* Camellia sinensis* plant and is commonly used as beverage worldwide. Although green tea shows vital role in diseases control, exact mechanism of action is still under investigation. The possible mechanisms of action of green tea in cancer prevention and progression are as follows:

- Green tea act as inhibitor of cyclooxygenase, lipoxygenases, tumour necrosis factor, and interleukin pathways and ultimately controls the development and progression of tumour.
- Green tea shows chemopreventive effect via activation of tumour suppressor genes such as p53 and PTEN/p21, regulation of apoptosis (bcl2/Bax), and inhibition of angiogenesis and other transcription factors involved in the cancer development and progression.
- It shows role in neutralization of free radical and damage of macromolecules due to high antioxidant capacity and finally prevents the pathogenesis of tumour.
- Another possible mechanism of green tea in cancer prevention is through the modulation of genes involved in initiations, promotion, and progression of tumour (Figure 2).

## 4. Anticancer Effect of Green Tea through Modulation of Cell Signalling Pathways

**Figure 3:**
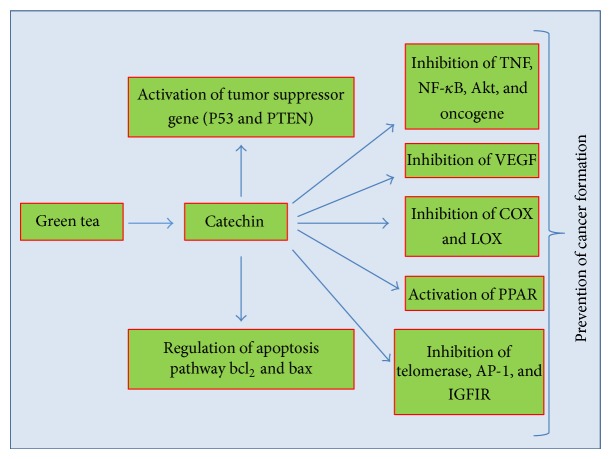
Green tea modulates the various cell signalling pathways in cancer.

Cancer is multifactorial disease including genetic and metabolic alterations. The current mode of treatment based on allopath is expensive and also alter the various cell signalling pathways. The diseases management based on natural products especially plants sources is inexpensive and shows fewer side effects. In this view, various medicinal plants/natural products have shown effect as anticancer via modulation of cell signalling pathways and other biological activities [17–21]. Earlier finding based on clinical trials and animal model has confirmed that green tea and its constituents play an important role in cancer prevention via activation/inactivation of genetic pathways. The role of green tea in cancer prevention and treatment based on modulation of cell signalling pathways is discussed in detail below (Figure 3).

### 4.1. Effect of Green Tea on Tumour Suppressor Gene

Tumour suppressor gene p53 is the guardian of all genes and regulates the various other molecular pathways. Altered expression/inactivation of tumour suppressor gene has been observed in various types of tumours. In this view, green tea and its constituents show a significant role in the activation of p53 gene (Figure 3).

A study demonstrated that GTP and EGCG increase p53 transcriptional activity and acetylation by suppressing class I histone deacetylases [22] and other studies showed EGCG-induced p53-dependent apoptosis in cancer cells [23].

EGCG, a chief constituent of green tea, induced the expression of p53 and its targets p21 and Bax in prostate cancer cells with wild-type but not inactive p53 [24] and other findings demonstrate that EGCG also illustrate role in the activation of p53 and Bax in breast cancer cells [25]. Treatment of cultured MCF-7 cells with green tea constituents such as EGCG increased the ratio of hypo- to hyperphosphorylated Rb, and this treatment also increased the levels of other proteins such as p53, p21 (waf1/CIP1), and p27 [26]. Other investigators have confirmed that EGCG at 10 to 40 *μ*mol/L induces p53-dependent apoptosis of JB6 cells through mitochondrial death pathway [27].

PTEN (phosphatase and tensin homolog deleted on chromosome TEN) and P16 are other types of tumor suppressor gene and altered expression of these genes has been noticed in tumours. An important finding revealed that epigallocatechin-3-gallate altered the p16 methylation pattern from methylated to unmethylated as a result of folic acid deprivation [28].

Another study has confirmed that EGCG treatment demonstrated a significant and dose-dependent inhibition of PDK1 and PTEN phosphorylation and also proposes that Akt activation by EGCG in A549 cells occurs neither through PDK1 activation nor through PTEN inactivation [29]. Other study findings confirmed that Epigallocatechin gallate (EGCG) and sulforaphane (SFN) combinations have tendency to enhance cisplatin-induced apoptosis and G2/M phase arrest via upregulation of p21, thus enhancing the efficacy of cisplatin on both cisplatin-sensitive and cisplatin-resistant ovarian cancer cells [30].

### 4.2. Effect of Green Tea on Apoptosis

Apoptosis is a process to keep up the normal and healthy internal milieu. Altered ratio of bcl2 and bax has been noticed in different types of cancer. Therefore, regulation of apoptosis process is critical step in the prevention of cancer.* In vitro* based study has shown that green tea extract, especially its major polyphenolic component (−)-epigallocatechin-3-gallate, is capable of inhibiting the growth of a variety of mouse and human cancer cells via the induction of apoptosis [31–33]. Other investigators have reported that (−)-Epigallocatechin-3-gallate rapidly induced apoptotic cell death in various malignant B-cell lines in a dose- and time-dependent manner [34]. Chemopreventive/antiproliferative potential of (−)-epigallocatechin gallate (EGCG) was checked in T24 human bladder cancer cell line and it was observed that EGCG treatment caused dose- and time-dependent inhibition of cellular proliferation and cell viability and induced apoptosis [35]. A finding showed that green tea and its constituents selectively induce apoptosis in oral carcinoma cells, while EGCG was able to inhibit the growth and invasion of oral carcinoma [36]. A significant result revealed that green tea polyphenols induce the transcription of* p21/waf1* and* Bax* and also enhance proteasomal degradation of class I HDACs and increase acetylation of histone H3 that lead to cell cycle arrest and apoptosis in prostate cancer cells [37].

Study was performed to investigate the effect of green tea polyphenols and chief constituent such as epigallocatechin-3-gallate, on the induction of apoptosis and regulation of cell cycle in human and mouse carcinoma cells and study results concluded that green tea may protect against cancer by causing cell cycle arrest and inducing apoptosis [38]. A vital review presented the available scientific literature about the effects of green tea polyphenols and its chief constituents EGCG on signaling pathways in prostate cancer [39].

### 4.3. Effects of Green Tea on Angiogenesis

Angiogenesis plays an important role in the tumour development and progression. Inhibition of angiogenesis is the most important step in prevention and treatment of cancer. Previous studies showed that treating nude mice with EGCG, constituents of green tea resulted in noticeable inhibition of growth, vascularity, and proliferation of human colon cancer xenografts [40].

EGCG, constituent of green tea, significantly shows a role in the inhibition of VEGF expression by suppressing the activation of HIF-1*α* and NF-*κ*B pathways, thereby inhibiting tumor growth, proliferation, migration, and angiogenesis of breast cancer [41].

Earlier investigators reported that that EGCG, a major polyphenol of green tea inhibited angiogenesis through blocking Erk-1 and Erk-2 activation and VEGF expression [40].

### 4.4. Effects of Green Tea on Phase I and Phase II Enzymes

Phase I and phase II genes/enzymes play role in modulation of invasion and progression of tumour. However, enzymes such as CYP 450 and GST have important role in the cancer via inhibition or activation of initiation, promotion, and progression process. However, modulations of CYP450 and GST enzymes/genes are one of the vital points in management of cancer development and progression. Green tea and its constituents show dual role in this phenomena via inhibition of CYP and activation of GST genes (Figure 3). An experimentation was performed to investigate the effect of green tea polyphenols (GTP) on the activities of phase I and phase II enzymes and results revealed supplementation of GTP by both simultaneous and posttreatment mode (200 mg/kg) and there was a significant increase in the activity of GST and UDP-GT and a significant decrease in the activity of phase I enzymes [42].

### 4.5. Effect of Green Tea on Cyclooxygenase and Lipoxygenase

Cyclooxygenase (COX) is also known as prostaglandin (PG) H synthase and catalyses the stages of prostanoids synthesis [43]. Altered cyclooxygenase/lipoxygenase has been noticed in various tumours. An important finding has examined the inhibitory effects of EGCG on signaling pathways controlling COX-2 expression and effect of EGCG on COX-2 expression noticed through decreased COX-2 promoter activity via inhibition of nuclear factor kappaB (NF-kappaB) activation [44].

The effects of green and black tea polyphenols on cyclooxygenase (COX) and lipoxygenase (LOX) were studied and revealed that at a concentration of 30 microg/mL, (−)-epigallocatechin-3-gallate (EGCG), (−)-epigallocatechin (EGC), and (−)-epicatechin-3-gallate (ECG) from green tea and theaflavins from black tea inhibited LOX-dependent activity by 30–75% [45]. An experimentation was designed to evaluate the effects of green tea and a high-fat diet on arachidonic acid metabolism and aberrant crypt foci formation in an azoxymethane- (AOM-) induced colon carcinogenesis mouse model and results revealed that consumption of green tea and dietary fat modulates 5-lipoxygenase-dependent pathway of arachidonic acid metabolism during AOM-induced colon carcinogenesis [46].

Another important study has demonstrated that pretreatment of the green tea extract enriched with catechin and epigallocatechin gallate showed inhibition of COX-2 expression induced through the tumor promoter 12-O-tetradecanoylphorbol-13-acetate (TPA) in mouse skin [47].

### 4.6. Effect of Green Tea on Akt Pathways

Akt/PIP3 pathways show an important role in the development and progression of tumour. EGCG, a key component of green tea, inhibited the basal activation of phospho-Akt and Akt kinase activity after 30 min of treatment; the inhibition of Akt kinase activity by EGCG preceded the suppression of surviving after 1-hour treatment and followed the increased caspase-9 activity after 6 h treatment [48]. T24 human bladder cancer cell line based test was performed to evaluate the chemopreventive/antiproliferative potential of EGCG and results showed that EGCG inhibits phosphatidylinositol 3′-kinase/Akt activation that in turn shows role in the modulation of Bcl-2 family proteins, leading to enhanced apoptosis of T24 cells [49]. Experiment was performed to examine the effects of EGCG on vascular endothelial growth factor (VEGF) production by YCU-H891 HNSCC and MDA-MB-231 breast carcinoma cell lines and found that treatment with EGCG inhibited the constitutive activation of the EGFR, Stat3, and Akt in both cell lines [50].

### 4.7. Effect of Green Tea on HER2/HER3 and EGFR Pathway

An oncogene is a mutated gene that shows an important role in the pathogenesis of diseases including cancer. Activation or overexpression of ErbB such as ErbB2 (HER2) and ErbB3 (HER3) and EGFR has been observed in various types of tumours. Green tea inhibits the growth of cancer cells by inhibiting the activation of HER2 and HER3. An important study was carried out to observe the effects of epigallocatechin-3 gallate (EGCG) on HER2/neu-overexpressing breast cancer cells and results demonstrated that EGCG reduced signaling through the phosphatidylinositol 3-kinase, Akt kinase to NF-kappaB pathway because of inhibition of basal HER2/neu receptor tyrosine phosphorylation [51]. Another study was performed to examine the effects of EGCG on activation of the HER2 receptor in human HNSCC and breast carcinoma cell lines and results confirmed that treatment of human HNSCC and breast carcinoma cell lines with 10 or 30 *μ*g of EGCG causes 50% inhibition of growth, noticeably inhibiting the phosphorylation of HER2 in both cell lines [52]. An experiment was performed to evaluate the effects of EGCG, a chief constituent of green tea on the HER3 RTK and on COX-2 expression in the SW837 human colon cancer cell line and results showed that treatment of SW837 colon cancer cells with 20 *μ*g/mL of EGCG caused decrease in the phosphorylated forms of EGFR, HER2, and HER3 within 6 hours of treatment [53].

Elevated levels of the epidermal growth factor receptor (EGFR) have been noticed in several of tumour. Various medicinal plants have confirmed the inhibitory effect on cancer progression via inhibiting the activities of EGFR. A test was made to investigate the effects of EGCG on the proliferation of human epidermoid carcinoma cell line, A431, and results revealed that EGCG strongly inhibited the protein tyrosine kinase (PTK) activities of EGF-R, PDGF-R, and FGF-R [54]. Another experiment was performed to examine the effects of EGCG on cellular localization of the EGFR in cells such as SW480 cells and results of the study demonstrated that treatment of the cells for 30 min with 1 *μ*g/mL of EGCG caused a decrease in cell surface-associated EGFRs and this was associated with internalization of EGFRs into endosomal vesicles [55]. Experiment was carried out to evaluate the effects of EGCG (10–100 *μ*g/mL) treatment on growth and invasion in a breast carcinoma cell line resistant to tamoxifen (MCF-7Tam) and parental MCF-7 and the results revealed that dose-dependent downregulation of EGFR mRNA expression and protein level occurred after 50 *μ*g/mL EGCG treatments of MCF-7Tam cells [56].

### 4.8. Effect of Green Tea on c-Met and PDGFR

Activation of the other member of receptor tyrosine kinases (RTKs) including c-Met and PDGFR is also suppressed by green tea catechins, and this is associated with cancer prevention. An important study results confirmed that EGCG, a chief tea polyphenol, inhibited cell proliferation in erlotinib-sensitive and -resistant cell lines, including those with c-Met overexpression, and acquired resistance to erlotinib and furthermore also completely inhibited ligand-induced c-Met phosphorylation and partially inhibited EGFR phosphorylation [57].

Overactivity of PDGF has been associated with cancer development and progression and also shows role in the pathogenesis of various diseases. An important result demonstrated that PDGF-induced mRNA expression of c-fos and egr-1 was totally inhibited in EGCG-treated vascular SMCs [58].

### 4.9. Effect of Green Tea on MAPK/RAS Pathways

Altered activity of MAPK/RAS pathways is the one of the culprits in the cancer development and progression. Natural inhibitors show important therapeutic role in the regulation of the activity of MAPK/RAS pathways and finally prevent the carcinogenesis. Green tea and its constituents have shown role as inhibitor of the activity of mitogen-activated protein kinases (MAPKs), key factor for survival signalling.

Prior investigation has reported that tea polyphenols EGCG and TFs were shown to decrease the activity of AP-1, a transcription factor via inhibition of MAPK, mainly the JNK in JB6 cells [59]. Other important findings revealed that polyphenols of tea such as EGCG with 10–20 *μ*g/mL inhibited MAPK pathway as well as activator protein-1 (AP-1) activity in human colon cancer cells [60]. A study concluded that reduction of the AR and growth factor IGF-1, modulation of inflammation biomarkers, and decrease in the MAPK signaling may contribute to the reduction in cell proliferation and apoptosis induction and therefore provide a biochemical basis of EGCG suppressing prostate cancer without toxicity [61].

Other finding results have shown that lung tissue of the mice treated with tobacco-specific nitrosamine 4-(methylnitrosamine)-1-(3-pyridyl)-1-butanone NNK showed a significantly high level of expression in c-myc, c-raf, and c-H-ras oncogenes after 4 or 8 weeks, and they were all inhibited by 2% tea drinking with inhibitory rates of 50%, 20%, and 50%, respectively [62].

### 4.10. Effect of Green Tea on Androgen Receptor

Overexpression of androgen and its receptor has been noticed in various tumors. Natural products and its constituents show role in the cancer prevention via downregulation of various genes including degradation of androgen. Earlier results have shown that EGCG, chief constituents of green tea, was observed subsequently to inhibit nuclear translocation and protein expression of AR in a tumor xenograft model [63].

Another study revealed that EGCG suppressed cell proliferation, prostate specific antigen (PSA) expression, and AR transcriptional activity in the different LNCaP sublines [64] and an important result has shown that tea polyphenol, EGCG, inhibited LNCaP cell growth and the expression of androgen regulated PSA and hK2 genes [65].

### 4.11. Effect of Green Tea on Nuclear Transcription Factor NF-*κ*B

Overexpression/altered nuclear transcription shows role in the development and progression of tumour and also shows alterations of other genetic pathways. Therefore, inhibition of nuclear transcription factor is a vital step in the management of tumour. A vital study results showed that EGCG, a chief green tea polyphenol, decreased lipopolysaccharide (LPS)-induced TNF*α* production in a dose-dependent fashion in the macrophage cell line, RAW264.7 and also inhibited LPS-induced TNF*α* mRNA expression as well as nuclear NF-*κ*B–binding activity [66].

Another significant finding showed that EGCG, the chief polyphenol of green tea, attenuated the excessive expression of CTGF induced by abdominal aortic constriction (AAC) or AngII and also showed role in the reduction of nuclear translocation of NF-*κ*B p65 subunit and degradation of I*κ*B-*α* [67]. Previous finding reported significant inhibitions of tumor growth and tumor angiogenesis through EGCG; in addition to that, a major green tea catechin inhibited the activation of HIF-1*α* and NF-*κ*B and decreased VEGF expression in breast carcinoma cells [68].

### 4.12. Effect of Green Tea on AP-1 Transcription Factor

AP-1 is a transcription factor that includes Jun and Fos protein families and play role in the cancer development and progression. Altered expression of AP-1 or Jun and Fos has been noticed in various tumours. Green tea ingredients play a role in cancer prevention through the inhibition of AP-1 transcription factor. Theaflavins and EGCG, chief constituents of green tea, inhibited UVB-induced AP-1 activation in a concentration-dependent manner [69]. A study was performed to investigate the antitumor promotion effects of EGCG and theaflavins and results showed that dose range of 5–20 *μ*M inhibited cell transformation; EGCG and theaflavins also inhibited AP-1-dependent transcriptional activity and DNA binding activity [59]. Another finding also showed that all of polyphenols of green tea and black tea except (−)-epicatechin showed strong inhibition of cell growth and AP-1 activity [70].

### 4.13. Effect of Green Tea on Signal Transducer and Activator of Transcription3 (Stat3)

Molecular effects of EGCG on human HNSCC cell lines such as YCU-N861 and YCU-H891 were examined and results of this study showed that treatment with EGCG inhibited phosphorylation of the EGFR, signal transducer, activator of transcription3 (Stat3), and extracellular regulated kinase (ERK) proteins [71].

Another study was performed to examine the effects of EGCG on vascular endothelial growth factor (VEGF) production by YCU-H891 HNSCC and MDA-MB-231 breast carcinoma cell lines and results confirm the constitutive activation of the EGFR, Stat3, and Akt inhibited by the treatment with a major biologically active component of green tea, EGCG [50].

### 4.14. Effect of Green Tea on Peroxisome Proliferator-Activated Receptors

Peroxisome proliferator-activated receptors (PPARs) belong to the superfamily of nuclear receptors [72]. It contains three genes that give PPAR-*α*, PPAR-*δ*, and PPAR-gamma different subtypes. Role of PPARs has been noticed in various tumors but the exact mechanism is not fully understood. In this vista, several natural products show a pivotal role in the activation of PPARs through the modulation of other genetic pathways and finally exhibit role in cancer prevention. Green tea and its constituents confirmed the diseases preventive role in various types of diseases. An important study results showed that moderate green tea extract concentration, supplemented to the cardiomyocyte medium from initial seeding, selectively activated the PPAR-beta/delta isoform [73] and other results confirmed that PPAR*α* is a direct negative regulator of heme oxygenase (HO-1) activation by EGCG and confers cell susceptibility to EGCG [74].

### 4.15. Effect of Green Tea on Telomerase

Telomeres are structures present at the ends of human chromosomes and show role in the protection and DNA damage. Upregulation of telomerase has been observed in several types of tumours. In this vista, green tea shows a significant role in the management of telomerase activity and consequently inhibits the tumour development and progression.

A valuable study was performed to examine the effects of epigallocatechin-3-gallate (EGCG) on human SCLC cells and results confirm that drug-sensitive (H69) and drug-resistant (H69VP) small-cell lung carcinoma cells incubation in EGCG at 1 × IC(50) for 24 h resulted in 50–60% reduced telomerase activity [75].

Previous study was carried out to investigate the effect of the major tea polyphenol, epigallocatechin gallate (EGCG), in cervical carcinogenesis and results showed that inhibited telomerase activity in human papillomavirus type 18- (HPV 18-) immortalized endocervical cell (HEN-18) and ectocervical cell (HEC-18), as well as serum-adapted HEN-18 (HEN-18S), transformed HEC-18 (HEN-18T) [76]. The* in vitro* activity measurement on cell lysates from U937 or HT29 cells showed that EGCG is the strongest telomerase inhibitor among the different catechins tested [77].

### 4.16. Effect of Green Tea on Insulin-Like Growth Factor I Receptor (IGFIR)

The insulin-like growth factor I receptor (IGFIR) shows a pivotal role in the development and progression of cancer. However, various medicinal plants or natural products have confirmed the inhibitory effect on insulin-like growth factor I receptor (IGFIR) protein and prevent the cancer development and progression. The insulin-like growth factor I receptor (IGFIR) is constitutively activated in Ewing family tumors (EFTs) and (−)-epigallocatechin gallate (EGCG), a chief constituents green tea, inhibits cell proliferation and survival of EFT cells through the inhibition of IGFIR activity and also EGCG treated EFT cell lines blocked the autophosphorylation of IGFIR tyrosine residues [78].

The treatment of SW837 colon cancer cell lines with 20 *μ*g/mL of EGCG caused decrease in the phosphorylated form of the IGF-1R protein within 6 h and when SW837 cells were treated with EGCG for 96 h with concentration 1.0 *μ*g/mL of EGCG, it also caused inhibition of activation of IGF-1R and decrease in the IGF-1 protein [79]. Effects of EGCG on activity of IGF/IGF-1R axis in HepG2 human hepatocellular carcinoma cells was determined and results confirmed that treatment of HepG2 cells with EGCG-induced apoptosis caused a decrease in the p-IGF-1R protein [80].

## 5. Safety and Toxicities of Green Tea

Green tea and its constituents play an important role in the maintenance of health due to their versatile therapeutic approach. Numerous results based on* in vivo* and* in vitro* study confirmed that green tea and their constituents such as EGCG show health promoting effect at certain dose through the modulation of various biological activities. On the other hand, green tea/EGCG overdose cause adverse complications including acute liver failure/hepatotoxicity via alterations in liver enzymes. An experiment based on rat model revealed that oral dose delivering 2000 mg EGCG preparation/kg was lethal to rats; but dose with 200 mg EGCG/kg induced no toxicity [81]. Other studies based on animal models have shown that green tea prevent hepatotoxicity induced by hepatotoxicants [82–84].

Recent finding results concluded that supplementation of 500 mg green tea polyphenols daily to postmenopausal osteopenic women for 24 weeks did not cause any adverse complications on liver and kidney function and had no influence on quality of life [85]. Earlier important results suggest high concentrations of green tea extract exert acute toxicity in rat liver cells and (−)-epigallocatechin-3-gallate, a chief constituents of green tea, seems to be one of the principal constituents responsible for such effect [86] and p.o. administration of EGCG or polyphenon E with daily dose of 800 mg (based on the EGCG content) for 4 weeks is safe and well tolerated in healthy human [87].

Experiment based on rat model showed that green tea extract with dose of 5% in diet for 13 wk induced thyroid enlargement in normal rats [88, 89] and other investigators studied the hepatotoxic effects of high dose EGCG, tea polyphenols, in male CF-1 mice and results indicated that higher bolus doses of EGCG are hepatotoxic to mice [90]. Numerous previous findings reported that patients show hepatotoxicity due to the consumption of supplements containing green tea extracts [91–95]. Latest data suggest that catechins are commonly present in several herbal dietary supplements (HDS) that are implicated in hepatotoxicity, even when not identified on the product label [96] and cases of hepatotoxicity have been linked with consumption of high doses green tea-containing supplements [97].

## 6. Bioavailability of Green Tea

The quantity of drugs that are accessible to the target tissues/or organ is important for biological activity. Highly polar drugs/fat soluble have low bioavailability due to incomplete absorption of drugs in the GI tract, poor absorption, and high rate of metabolism. Various earlier reports confirm that green tea constituents were found to be low in plasma than the actual ingested quantity and therefore it shows less biological activities. Earlier study proved that EGCG and EGC levels detected in plasma correspond to 0.2–2.0% of the ingested amount [98]. Various types of formulation based on nanoparticles, micelles, and liposome play an important role in the enhancement of absorption of green tea constituents and encapsulated form shows better results in modulation of biological activities due to high intestinal absorption. In this vista, an important study exhibited that encapsulation of catechins in chitosan nanoparticles (CS NP) enhances their intestinal absorption and is a promising strategy for improving their bioavailability [99].

Another study on basal cell carcinoma has shown that nearly no drug molecules were observed when free EGCG was administrated to BCCs, whereas EGCG encapsulated in liposomes with deoxycholic acid (DA) and ethanol increased drug deposition by 20-fold as compared to the free form [100]. It was previously proved that high efficiency and yield were achieved from the incorporation of catechin or EGCG inside the liposome structure [101]. Bioavailability of green tea differs with types of polyphenols. EGCG, chief polyphenols of green tea, is found in large proportion in plasma in a free form [102–104] and epigallocatechin gallate (EGCG), principal ingredients with dose of 50 mg, peak plasma concentrations were approximately 0.15 *μ*mol/L [105]. An important study reported that, at similar concentration of epigallocatechin and epigallocatechin-3-gallate in a green tea drink, plasma concentration of epigallocatechin concentration was observed approximately 2-3 times more than the epigallocatechin-3-gallate concentration after tea consumption [103].

## 7. Clinical Trials Based Study on Green Tea

Numerous series of preclinical studies have proven that green tea and their constituents show an important role in prevention and treatment of human cancers. A study based on 49,920 men subjects in Japan aged 40–69 years who completed a questionnaire that included green tea consumption habit at baseline and were followed until the end of 2004 established that drinking 5 or more cups/day compared with less than 1 cup/day demonstrated decreased risk of advanced prostate cancer (multivariate relative risk was 0.52, 95% confidence interval: 0.28, 0.96) [106]. Another important study has investigated antineoplastic effects of green tea in patients with androgen independent prostate carcinoma; 42 patients who were asymptomatic and had manifested progressive prostate specific antigen elevation with hormone therapy were checked and study results concluded that green tea carries limited antineoplastic activity, as defined by a decline in PSA levels, among patients with androgen independent prostate carcinoma [107]. A significant and first study confirmed that green tea catechins have potent* in vivo* chemoprevention activity for human prostate cancer and data of this study suggest that up to 90% of chemoprevention efficacy can be obtained by GTCs administration in men prone to develop prostate cancer [108] and study reports indicate that human metachronous colorectal adenomas were significantly prevented by daily green tea consumption supplemented with GTE [109].

## 8. Conclusion

The allopath based anticancer drugs are expensive and also show adverse effect via alteration in molecular pathways. Green tea is widely used in traditional medicine since ancient time due to being inexpensive, efficacy, and fewer side effect properties. The studies based on animal model and cell lines demonstrated anticancer activity of green tea and its constituents by modulating cell signalling pathways including angiogenesis, apoptosis, and transcription factor. Extensive study based on animal model may contribute to understanding the exact mechanism of action and toxicity level/side effect without alteration of therapeutic potential. Additionally, the detailed study of green tea and its constituents based on clinical trials would be very helpful in the designing of novel anticancer drugs.

## References

[1] Al-Bukhari M. I., Al-Bukhari S. (1976). *The Collection of Authentic Sayings of Prophet Mohammad (Peace Be Upon Him)*.

[2] Ahmad A., Husain A., Mujeeb M. (2013). A review on therapeutic potential of Nigella sativa: a miracle herb. *Asian Pacific Journal of Tropical Biomedicine*.

[3] Rahmani A. H., Aly S. M., Ali H., Babiker A. Y., Suikar S., Khan A. A. (2014). Therapeutic effects of date fruits (*Phoenix dactylifera*) in the prevention of diseases via modulation of anti-inflammatory, anti-oxidant and anti-tumour activity. *International Journal of Clinical and Experimental Medicine*.

[4] Rahmani A. H., Albutti A. S., Aly S. M. (2014). Therapeutics role of olive fruits/oil in the prevention of diseases via modulation of anti-oxidant, anti-tumour and genetic activity. *International Journal of Clinical and Experimental Medicine*.

[5] Khan M. A., Chen H.-C., Tania M., Zhang D.-Z. (2011). Anticancer activities of *Nigella sativa* (black cumin). *African Journal of Traditional, Complementary, and Alternative Medicines*.

[6] Khan N., Mukhtar H. (2007). Tea polyphenols for health promotion. *Life Sciences*.

[7] McKay D. L., Blumberg J. B. (2002). The role of tea in human health: an update. *Journal of the American College of Nutrition*.

[8] Khan N., Afaq F., Saleem M., Ahmad N., Mukhtar H. (2006). Targeting multiple signaling pathways by green tea polyphenol (−)-epigallocatechin-3-gallate. *Cancer Research*.

[9] Yanaga H., Fujii T., Koga T., Araki R., Shirouzu K. (2002). Prevention of carcinogenesis of mouse mammary epithelial cells RIII/MG by epigallocatechin gallate. *International Journal of Molecular Medicine*.

[10] Mineva N. D., Paulson K. E., Naber S. P., Yee A. S., Sonenshein G. E. (2013). Epigallocatechin-3-gallate inhibits stem-like inflammatory breast cancer cells. *PloS ONE*.

[11] Schramm L. (2013). Going green: the role of the green tea component EGCG in chemoprevention. *Journal of Carcinogenesis &; Mutagenesis*.

[12] Balentine D. A., Wiseman S. A., Bouwens L. C. M. (1997). The chemistry of tea flavonoids. *Critical Reviews in Food Science and Nutrition*.

[13] Dufresne C. J., Farnworth E. R. (2001). A review of latest research findings on the health promotion properties of tea. *Journal of Nutritional Biochemistry*.

[14] Higdon J. V., Frei B. (2003). Tea catechins and polyphenols: health effects, metabolism, and antioxidant functions. *Critical Reviews in Food Science and Nutrition*.

[15] Mukhtar H., Ahmad N. (2000). Tea polyphenols: prevention of cancer and optimizing health. *The American Journal of Clinical Nutrition*.

[16] Harbowy M. E., Balentine D. A., Davies A. P., Cai Y. (1997). Tea chemistry. *Critical Reviews in Plant Sciences*.

[17] Rahmani A. H., Al Zohairy M. A., Aly S. M., Khan M. A. (2014). Curcumin: a potential candidate in prevention of cancer via modulation of molecular pathways. *BioMed Research International*.

[18] Randhawa M. A., Alghamdi M. S. (2011). Anticancer activity of *Nigella sativa* (Black Seed)—a review. *The American Journal of Chinese Medicine*.

[19] Rahmani A. H., Al Shabrmi F. M., Aly S. M. (2014). Active ingredients of ginger as potential candidates in the prevention and treatment of diseases via modulation of biological activities. *International Journal of Physiology, Pathophysiology and Pharmacology*.

[20] Balentine D. A., Wiseman S. A., Bouwens L. C. (1997). The chemistry of tea flavonoids. *Critical Reviews in Food Science and Nutrition*.

[21] Bhanot A., Sharma R., Noolvi M. N. (2011). Natural sources as potential anti-cancer agents: a review. *International Journal of Phytomedicine*.

[22] Thakur V. S., Gupta K., Gupta S. (2012). Green tea polyphenols increase p53 transcriptional activity and acetylation by suppressing class I histone deacetylases. *International Journal of Oncology*.

[23] Hastak K., Gupta S., Ahmad N., Agarwal M. K., Agarwal M. L., Mukhtar H. (2003). Role of p53 and NF-*κ*B in epigallocatechin-3-gallate-induced apoptosis of LNCaP cells. *Oncogene*.

[24] Hastak K., Agarwal M. K., Mukhtar H., Agarwal M. L. (2005). Ablation of either p21 or Bax prevents p53-dependent apoptosis induced by green tea polyphenol epigallocatechin-3-gallate. *The FASEB Journal*.

[25] Roy A. M., Baliga M. S., Katiyar S. K. (2005). Epigallocatechin-3-gallate induces apoptosis in estrogen receptor-negative human breast carcinoma cells via modulation in protein expresssion of p53 and Bax and caspase-3 activation. *Molecular Cancer Therapeutics*.

[26] Liang Y.-C., Lin-Shiau S.-Y., Chen C.-F., Lin J.-K. (1999). Inhibition of cyclin-dependent kinases 2 and 4 activities as well as induction of Cdk inhibitors p21 and p27 during growth arrest of human breast carcinoma cells by (−)-epigallocatechin-3-gallate. *Journal of Cellular Biochemistry*.

[27] Qin J., Chen H.-G., Yan Q. (2008). Protein phosphatase-2A is a target of epigallocatechin-3-gallate and modulates p53-Bak apoptotic pathway. *Cancer Research*.

[28] Navarro-Perán E., Cabezas-Herrera J., Campo L. S. D., Rodríguez-López J. N. (2007). Effects of folate cycle disruption by the green tea polyphenol epigallocatechin-3-gallate. *International Journal of Biochemistry and Cell Biology*.

[29] Kim M. J., Kim H. I., Chung J., Jeong T. S., Park H. R. (2009). (−)-Epigallocatechin-3-gallate (EGCG) increases the viability of serum-starved A549 cells through its effect on Akt. *American Journal of Chinese Medicine*.

[30] Chen H., Landen C. N., Li Y., Alvarez R. D., Tollefsbol T. O. (2013). Enhancement of cisplatin-mediated apoptosis in ovarian cancer cells through potentiating G2/M arrest and p21 upregulation by combinatorial epigallocatechin gallate and sulforaphane. *Journal of Oncology*.

[31] Yang C. S., Wang Z.-Y. (1993). Tea and cancer. *Journal of the National Cancer Institute*.

[32] Gupta S., Ahmad N., Nieminen A.-L., Mukhtar H. (2000). Growth inhibition, cell-cycle dysregulation, and induction of apoptosis by green tea constituent (-)-epigallocatechin-3-gallate in androgen-sensitive and androgen-insensitive human prostate carcinoma cells. *Toxicology and Applied Pharmacology*.

[33] Barthelman M., Bair W. B., Stickland K. K. (1998). (−)-Epigallocatechin-3-gallate inhibition of ultraviolet B-induced AP-1 activity. *Carcinogenesis*.

[34] Nakazato T., Ito K., Ikeda Y., Kizaki M. (2005). Green tea component, catechin, induces apoptosis of human malignant B cells via production of reactive oxygen species. *Clinical Cancer Research*.

[35] Qin J., Xie L.-P., Zheng X.-Y. (2007). A component of green tea, (−)-epigallocatechin-3-gallate, promotes apoptosis in T24 human bladder cancer cells via modulation of the PI3K/Akt pathway and Bcl-2 family proteins. *Biochemical and Biophysical Research Communications*.

[36] Hsu S. D., Singh B. B., Lewis J. B. (2002). Chemoprevention of oral cancer by green tea. *General dentistry*.

[37] Thakur V. S., Gupta K., Gupta S. (2012). Green tea polyphenols causes cell cycle arrest and apoptosis in prostate cancer cells by suppressing class I histone deacetylases. *Carcinogenesis*.

[38] Ahmad N., Feyes D. K., Nieminen A.-L., Agarwal R., Mukhtar H. (1997). Green tea constituent epigallocatechin-3-gallate and induction of apoptosis and cell cycle arrest in human carcinoma cells. *Journal of the National Cancer Institute*.

[39] Khan N., Mukhtar H. (2013). Modulation of signaling pathways in prostate cancer by green tea polyphenols. *Biochemical Pharmacology*.

[40] Jung Y. D., Kim M. S., Shin B. A. (2001). EGCG, a major component of green tea, inhibits tumour growth by inhibiting VEGF induction in human colon carcinoma cells. *British Journal of Cancer*.

[41] Gu J.-W., Makey K. L., Tucker K. B. (2013). EGCG, a major green tea catechin suppresses breast tumor angiogenesis and growth via inhibiting the activation of HIF-1*α* and NF*κ*B, and VEGF expression. *Vascular Cell*.

[42] Srinivasan P., Suchalatha S., Babu P. V. A. (2008). Chemopreventive and therapeutic modulation of green tea polyphenols on drug metabolizing enzymes in 4-nitroquinoline 1-oxide induced oral cancer. *Chemico-Biological Interactions*.

[43] Minghetti L. (2004). Cyclooxygenase-2 (COX-2) in inflammatory and degenerative brain diseases. *Journal of Neuropathology and Experimental Neurology*.

[44] Peng G., Dixon D. A., Muga S. J., Smith T. J., Wargovich M. J. (2006). Green tea polyphenol (−)-epigallocatechin-3-gallate inhibits cyclooxygenase-2 expression in colon carcinogenesis. *Molecular Carcinogenesis*.

[45] Hong J., Smith T. J., Ho C.-T., August D. A., Yang C. S. (2001). Effects of purified green and black tea polyphenols on cyclooxygenase- and lipoxygenase-dependent metabolism of arachidonic acid in human colon mucosa and colon tumor tissues. *Biochemical Pharmacology*.

[46] Ju J., Liu Y., Hong J., Huang M.-T., Conney A. H., Yang C. S. (2003). Effects of green tea and high-fat diet on arachidonic acid metabolism and aberrant crypt foci formation in an azoxymethane-induced colon carcinogenesis mouse model. *Nutrition and Cancer*.

[47] Kundu J. K., Na H. K., Chun K. S. (2003). Inhibition of phorbol ester-induced COX-2 expression by epigallocatechin gallate in mouse skin and cultured human mammary epithelial cells. *Journal of Nutrition*.

[48] Tang Y., Zhao D. Y., Elliott S. (2007). Epigallocatechin-3 gallate induces growth inhibition and apoptosis in human breast cancer cells through survivin suppression. *International Journal of Oncology*.

[49] Qin J., Xie L.-P., Zheng X.-Y. (2007). A component of green tea, (−)-epigallocatechin-3-gallate, promotes apoptosis in T24 human bladder cancer cells via modulation of the PI3K/Akt pathway and Bcl-2 family proteins. *Biochemical and Biophysical Research Communications*.

[50] Masuda M., Suzui M., Lim J. T. E., Deguchi A., Soh J.-W., Weinstein I. B. (2002). Epigallocatechin-3-gallate decreases VEGF production in head and neck and breast carcinoma cells by inhibiting EGFR-related pathways of signal transduction. *Journal of Experimental Therapeutics and Oncology*.

[51] Pianetti S., Guo S., Kavanagh K. T., Sonenshein G. E. (2002). Green tea polyphenol epigallocatechin-3 gallate inhibits Her-2/neu signaling, proliferation, and transformed phenotype of breast cancer cells. *Cancer Research*.

[52] Masuda M., Suzui M., Lim J. T. E., Weinstein I. B. (2003). Epigallocatechin-3-gallate inhibits activation of HER-2/neu and downstream signaling pathways in human head and neck and breast carcinoma cells. *Clinical Cancer Research*.

[53] Shimizu M., Deguchi A., Joe A. K., Mckoy J. F., Moriwaki H., Weinstein I. B. (2005). EGCG inhibits activation of HER3 and expression of cyclooxygenase-2 in human colon cancer cells. *Journal of Experimental Therapeutics and Oncology*.

[54] Liang Y.-C., Lin-shiau S.-Y., Chen C.-F., Lin J.-K. (1997). Suppression of extracellular signals and cell proliferation through EGF receptor binding by (-)-epigallocatechin gallate in human A431 epidermoid carcinoma cells. *Journal of Cellular Biochemistry*.

[55] Adachi S., Nagao T., To S. (2008). (−)-epigallocatechin gallate causes internalization of the epidermal growth factor receptor in human colon cancer cells. *Carcinogenesis*.

[56] Farabegoli F., Papi A., Orlandi M. (2011). (–)-Epigallocatechin-3-gallate down-regulates EGFR, MMP-2, MMP-9 and EMMPRIN and inhibits the invasion of MCF-7 tamoxifen-resistant cells. *Bioscience Reports*.

[57] Milligan S. A., Burke P., Coleman D. T. (2009). The green tea polyphenol EGCG potentiates the antiproliferative activity of c-Met and epidermal growth factor receptor inhibitors in non-small cell lung cancer cells. *Clinical Cancer Research*.

[58] Ahn H.-Y., Hadizadeh K. R., Seul C., Yun Y.-P., Vetter H., Sachinidis A. (1999). Epigallocathechin-3 gallate selectively inhibits the PDGF-BB-induced intracellular signaling transduction pathway in vascular smooth muscle cells and inhibits transformation of sis-transfected NIH 3T3 fibroblasts and human glioblastoma cells (A172). *Molecular Biology of the Cell*.

[59] Dong Z., Ma W.-Y., Huang C., Yang C. S. (1997). Inhibition of tumor promoter-induced activator protein 1 activation and cell transformation by tea polyphenols, (−)-epigallocatechin gallate, and theaflavins. *Cancer Research*.

[60] Shimizu M., Deguchi A., Lim J. T. E., Moriwaki H., Kopelovich L., Weinstein I. B. (2005). (-)-Epigallocatechin gallate and polyphenon E inhibit growth and activation of the epidermal growth factor receptor and human epidermal growth factor receptor-2 signaling pathways in human colon cancer cells. *Clinical Cancer Research*.

[61] Harper C. E., Patel B. B., Wang J., Eltoum I. A., Lamartiniere C. A. (2007). Epigallocatechin-3-gallate suppresses early stage, but not late stage prostate cancer inTRAMP mice: mechanisms of action. *Prostate*.

[62] Hu G., Han C., Chen J. (1995). Inhibition of oncogene expression by green tea and (-)-epigallocatechin gallate in mice. *Nutrition and Cancer*.

[63] Siddiqui I. A., Asim M., Hafeez B. B., Adhami V. M., Tarapore R. S., Mukhtar H. (2011). Green tea polyphenol EGCG blunts androgen receptor function in prostate cancer. *The FASEB Journal*.

[64] Chuu C.-P., Chen R.-Y., Kokontis J. M., Hiipakka R. A., Liao S. (2009). Suppression of androgen receptor signaling and prostate specific antigen expression by (-)-epigallocatechin-3-gallate in different progression stages of LNCaP prostate cancer cells. *Cancer Letters*.

[65] Ren F., Zhang S., Mitchell S. H., Butler R., Young C. Y. F. (2000). Tea polyphenols down-regulate the expression of the androgen receptor in LNCaP prostate cancer cells. *Oncogene*.

[66] Yang F., De Villiers W. J. S., McClain C. J., Varilek G. W. (1998). Green tea polyphenols block endotoxin-induced tumor necrosis factor- production and lethality in a murine model. *Journal of Nutrition*.

[67] Cai Y., Yu S.-S., Chen T.-T. (2013). EGCG inhibits CTGF expression via blocking NF-*κ*B activation in cardiac fibroblast. *Phytomedicine*.

[68] Gu J.-W., Makey K. L., Tucker K. B. (2013). EGCG, a major green tea catechin suppresses breast tumor angiogenesis and growth via inhibiting the activation of HIF-1*α* and NF*κ*B, and VEGF expression. *Vascular Cell*.

[69] Nomura M., Ma W.-Y., Huang C. (2000). Inhibition of ultraviolet B-induced AP-1 activation by theaflavins from black tea. *Molecular Carcinogenesis*.

[70] Chung J. Y., Huang C., Meng X., Dong Z., Yang C. S. (1999). Inhibition of activator protein 1 activity and cell growth by purified green tea and black tea polyphenols in H-ras-transformed cells: structure- activity relationship and mechanisms involved. *Cancer Research*.

[71] Masuda M., Suzui M., Weinstein I. B. (2001). Effects of epigallocatechin-3-gallate on growth, epidermal growth factor receptor signaling pathways, gene expression, and chemosensitivity in human head and neck squamous cell carcinoma cell lines. *Clinical Cancer Research*.

[72] Green S., Wahli W. (1994). Peroxisome proliferator-activated receptors: finding the orphan a home. *Molecular and Cellular Endocrinology*.

[73] Danesi F., di Nunzio M., Boschetti E., Bordoni A. (2009). Green tea extract selectively activates peroxisome proliferator-activated receptor beta/delta in cultured cardiomyocytes. *British Journal of Nutrition*.

[74] Zhang S., Yang X., Luo J. (2014). PPAR*α* activation sensitizes cancer cells to epigallocatechin-3- gallate (egcg) treatment via suppressing heme oxygenase-1. *Nutrition and Cancer*.

[75] Sadava D., Whitlock E., Kane S. E. (2007). The green tea polyphenol, epigallocatechin-3-gallate inhibits telomerase and induces apoptosis in drug-resistant lung cancer cells. *Biochemical and Biophysical Research Communications*.

[76] Yokoyama M., Noguchi M., Nakao Y., Pater A., Iwasaka T. (2004). The tea polyphenol, (−)-epigallocatechin gallate effects on growth, apoptosis, and telomerase activity in cervical cell lines. *Gynecologic Oncology*.

[77] Naasani I., Seimiya H., Tsuruo T. (1998). Telomerase inhibition, telomere shortening, and senescence of cancer cells by tea catechins. *Biochemical and Biophysical Research Communications*.

[78] Kang H.-G., Jenabi J. M., Liu X. F., Reynolds C. P., Triche T. J., Sorensen P. H. B. (2010). Inhibition of the insulin-like growth factor I receptor by epigallocatechin gallate blocks proliferation and induces the death of ewing tumor cells. *Molecular Cancer Therapeutics*.

[79] Shimizu M., Deguchi A., Hara Y., Moriwaki H., Weinstein I. B. (2005). EGCG inhibits activation of the insulin-like growth factor-1 receptor in human colon cancer cells. *Biochemical and Biophysical Research Communications*.

[80] Shimizu M., Shirakami Y., Sakai H. (2008). EGCG inhibits activation of the insulin-like growth factor (IGF)/IGF-1 receptor axis in human hepatocellular carcinoma cells. *Cancer Letters*.

[81] Isbrucker R. A., Edwards J. A., Wolz E., Davidovich A., Bausch J. (2006). Safety studies on epigallocatechin gallate (EGCG) preparations. Part 2: dermal, acute and short-term toxicity studies. *Food and Chemical Toxicology*.

[82] Sai K., Kai S., Umemura T. (1998). Protective effects of green tea on hepatotoxicity, oxidative DNA damage and cell proliferation in the rat liver induced by repeated oral administration of 2-nitropropane. *Food and Chemical Toxicology*.

[83] Chen J.-H., Tipoe G. L., Liong E. C. (2004). Green tea polyphenols prevent toxin-induced hepatotoxicity in mice by down-regulating inducible nitric oxide-derived prooxidants. *The American Journal of Clinical Nutrition*.

[84] Oz H. S., McClain C. J., Nagasawa H. T., Ray M. B., de Villiers W. J. S., Chen T. S. (2004). Diverse antioxidants protect against acetaminophen hepatotoxicity. *Journal of Biochemical and Molecular Toxicology*.

[85] Shen C.-L., Chyu M.-C., Pence B. C. (2010). Green tea polyphenols supplementation and Tai Chi exercise for postmenopausal osteopenic women: safety and quality of life report. *BMC Complementary and Alternative Medicine*.

[86] Schmidt M., Schmitz H.-J., Baumgart A. (2005). Toxicity of green tea extracts and their constituents in rat hepatocytes in primary culture. *Food and Chemical Toxicology*.

[87] Chow H.-H. S., Cai Y., Hakim I. A. (2003). Pharmacokinetics and safety of green tea polyphenols after multiple-dose administration of epigallocatechin gallate and polyphenon E in healthy individuals. *Clinical Cancer Research*.

[88] Sakamoto Y., Mikuriya H., Tayama K. (2001). Goitrogenic effects of green tea extract catechins by dietary administration in rats. *Archives of Toxicology*.

[89] Satoh K., Sakamoto Y., Ogata A. (2002). Inhibition of aromatase activity by green tea extract catechins and their endocrinological effects of oral administration in rats. *Food and Chemical Toxicology*.

[90] Lambert J. D., Kennett M. J., Sang S., Reuhl K. R., Ju J., Yang C. S. (2010). Hepatotoxicity of high oral dose (−)-epigallocatechin-3-gallate in mice. *Food and Chemical Toxicology*.

[91] Molinari M., Watt K. D. S., Kruszyna T. (2006). Acute liver failure induced by green tea extracts: case report and review of the literature. *Liver Transplantation*.

[92] Vial T., Bernard G., Lewden B., Dumortier J., Descotes J. (2003). Acute hepatitis due to Exolise, a Camellia sinensis-derived drug [1]. *Gastroenterologie Clinique et Biologique*.

[93] Pedrós C., Cereza G., García N., Laporte J.-R. (2003). Liver toxicity of Camellia sinensis dried atanolic extract. *Medicina Clinica*.

[94] Bonkovsky Md H. L. (2006). Hepatotoxicity associated with supplements containing Chinese green tea (Camellia sinensis). *Annals of Internal Medicine*.

[95] Dueñas Sadornil C., Fabregas Puigtió S., Durández R. (2004). Hepatotoxicity due to *Camellia sinensis*. *Medicina Clinica*.

[96] Navarro V. J., Bonkovsky H. L., Hwang S.-I., Vega M., Barnhart H., Serrano J. (2013). Catechins in dietary supplements and hepatotoxicity. *Digestive Diseases and Sciences*.

[97] Mazzanti G., Menniti-Ippolito F., Moro P. A. (2009). Hepatotoxicity from green tea: a review of the literature and two unpublished cases. *European Journal of Clinical Pharmacology*.

[98] Nakagawa K., Okuda S., Miyazawa T. (1997). Dose-dependent incorporation of tea catechins, (-)-epigallocatechin-3-gallate and (-)-epigallocatechin, into human plasma. *Bioscience, Biotechnology and Biochemistry*.

[99] Dube A., Nicolazzo J. A., Larson I. (2010). Chitosan nanoparticles enhance the intestinal absorption of the green tea catechins (+)-catechin and (−)-epigallocatechin gallate. *European Journal of Pharmaceutical Sciences*.

[100] Fang J. Y., Lee W. R., Shen S. C., Huang Y. L. (2006). Effect of liposome encapsulation of tea catechins on their accumulation in basal cell carcinomas. *Journal of Dermatological Science*.

[101] Rashidinejad A., Birch E. J., Sun-Waterhouse D., Everett D. W. (2014). Delivery of green tea catechin and epigallocatechin gallate in liposomes incorporated into low-fat hard cheese. *Food Chemistry*.

[102] Ullmann U., Haller J., Decourt J. P. (2003). A single ascending dose study of epigallocatechin gallate in healthy volunteers. *Journal of International Medical Research*.

[103] Meng X., Sang S., Zhu N. (2002). Identification and characterization of methylated and ring-fission metabolites of tea catechins formed in humans, mice, and rats. *Chemical Research in Toxicology*.

[104] Lee M.-J., Maliakal P., Chen L. (2002). Pharmacokinetics of tea catechins after ingestion of green tea and (-)-epigallocatechin-3-gallate by humans: Formation of different metabolites and individual variability. *Cancer Epidemiology Biomarkers and Prevention*.

[105] Manach C., Williamson G., Morand C., Scalbert A., Rémésy C. (2005). Bioavailability and bioefficacy of polyphenols in humans. I. Review of 97 bioavailability studies. *The American Journal of Clinical Nutrition*.

[106] Kurahashi N., Sasazuki S., Iwasaki M., Inoue M. (2008). Green tea consumption and prostate cancer risk in Japanese men: a prospective study. *The American Journal of Epidemiology*.

[107] Jatoi A., Ellison N., Burch P. A. (2003). A phase II trial of green tea in the treatment of patients with androgen independent metastatic prostate carcinoma. *Cancer*.

[108] Bettuzzi S., Brausi M., Rizzi F., Castagnetti G., Peracchia G., Corti A. (2006). Chemoprevention of human prostate cancer by oral administration of green tea catechins in volunteers with high-grade prostate intraepithelial neoplasia: a preliminary report from a one-year proof-of-principle study. *Cancer Research*.

[109] Shimizu M., Fukutomi Y., Ninomiya M. (2008). Green tea extracts for the prevention of metachronous colorectal adenomas: a pilot study. *Cancer Epidemiology Biomarkers and Prevention*.

